# Effects of ‘Target’ Plant Species Body Size on Neighbourhood Species Richness and Composition in Old-Field Vegetation

**DOI:** 10.1371/journal.pone.0082036

**Published:** 2013-12-13

**Authors:** Brandon S. Schamp, Lonnie W. Aarssen, Stephanie Wight

**Affiliations:** 1 Department of Biology, Algoma University, Sault Ste. Marie, Canada; 2 Department of Biology, Queen’s University, Kingston, Canada; University of Alberta, Canada

## Abstract

Competition is generally regarded as an important force in organizing the structure of vegetation, and evidence from several experimental studies of species mixtures suggests that larger mature plant size elicits a competitive advantage. However, these findings are at odds with the fact that large and small plant species generally coexist, and relatively smaller species are more common in virtually all plant communities. Here, we use replicates of ten relatively large old-field plant species to explore the competitive impact of target individual size on their surrounding neighbourhoods compared to nearby neighbourhoods of the same size that are not centred by a large target individual. While target individuals of the largest of our test species, *Centaurea jacea L.*, had a strong impact on neighbouring species, in general, target species size was a weak predictor of the number of other resident species growing within its immediate neighbourhood, as well as the number of resident species that were reproductive. Thus, the presence of a large competitor did not restrict the ability of neighbouring species to reproduce. Lastly, target species size did not have any impact on the species size structure of neighbouring species; i.e. they did not restrict smaller, supposedly poorer competitors, from growing and reproducing close by. Taken together, these results provide no support for a size-advantage in competition restricting local species richness or the ability of small species to coexist and successfully reproduce in the immediate neighbourhood of a large species.

## Introduction

According to traditional plant competition theory, relatively large species are generally expected to be better competitors, particularly for above-ground resources [1]–[3]. Evidence from several experimental studies of species mixtures is largely consistent with this ‘size-advantage’ hypothesis, with larger species generally suffering less growth suppression under experimentally ‘crowded’ conditions [4]–[12], and with above-ground competition being asymmetrical [13]. Although evidence has been mixed [12], [14], a recent meta-analysis also suggests that smaller competitor plants are at a disadvantage in below-ground competition [15]. Accordingly, within natural vegetation where the size of a species can approach its maximum potential (e.g., in the absence of grazing or resource stress), species with relatively large potential body size may be expected to limit the number of species–especially relatively small species–that can coexist with them.

The notion of a competitive size-advantage has been criticized, largely on the basis of casual observations that small plant species are ubiquitous and typically dominate (numerically) natural vegetation [16]. The experimental basis for a size-advantage relies on short-term experiments in which pairs of species compete from the seedling stage [4], [12] when competitive ranks have been observed to change depending on the relative age of competitors [17], [18]. Studies supporting a size-advantage have also measured the consequences of competition largely in terms of biomass reduction [19] while evidence suggests that seed production is more important in terms of defining competitive fitness [20].

Additionally, according to the ‘physical-space-niche’ (PSN) hypothesis [16], a smaller species–because of its smaller reproductive threshold size [21]–can reproduce successfully using fewer resources and with less physical space (thus defining the size of the PSN), while at the same time a larger species is less efficient at harvesting all of the resource units available within its larger PSN. Accordingly, within its own PSN, a mature plant of a larger species will leave more parcels of ‘left-over’ resource units that it cannot use, but which are sufficient in satisfying the PSN requirements of smaller species. In addition, the PSN hypothesis predicts that larger species not only generate these smaller PSN’s, but their physical presence also generates environmental heterogeneity through the variable impact of different large species on the small-scale physio-chemical (e.g. light and soil) environment [22]–[30]. Importantly, even if an individual of a small species must compete under crowded conditions for local resources with a suppressed or juvenile (i.e. small) individual offspring of a large neighbouring species, the former – because of its smaller PSN and greater ‘reproductive economy’ [16], [31] – is predicted nevertheless to successfully produce at least some offspring, while the latter (because of its larger PSN) is more likely to die leaving none [21]. Moreover, a locally dense collection of young establishing plants belonging to a large species will self-thin as the effects of competition accrue, thus leaving physical space ‘vacancies’ for smaller species to invade [32].

Recently, studies have begun to accumulate more evidence that a size advantage may not generally be realized within natural vegetation. Field studies, for example, have shown that the sizes of coexisting species are neither more similar nor more different than expected by random assembly–in both herbaceous [33] and woody/mixed vegetation [35], [36]–and that larger species in woody vegetation do not generally limit the number of species that can coexist within their immediate neighbourhoods [36]. These have been important contributions; however, additional studies, particularly in old-field vegetation which has provided most of the species examined in competition experiments, are required to definitively determine whether a size-advantage is functioning within natural vegetation. Additionally, it is possible that these old-field studies have focused on natural systems that are dominated by root competition, in which larger plants may or may not be expected to have an advantage [14], [15], [37].

Accordingly, in the present study we tested the following predictions associated with the ‘size-advantage’ hypothesis: Compared with randomly chosen neighbourhoods of equivalent area (not dominated by relatively large species), those that are centred by large species contain fewer resident species, fewer resident species that achieve reproduction, and particularly, fewer relatively small species.

## Materials and Methods

### Sites and Selection of ‘Target’ Species

Samples of relatively ‘big’ herbaceous plant (target) species were collected from old-field vegetation at Queen’s University Biological Station (QUBS) (44°33′N, 76°21′W) located north of Kingston, Ontario, Canada during the spring and summer of 2010. A candidate ‘big’ species was regarded as one that has a typical adult body size that is within about the top 20% of the body size of the resident species within a community, based on visual estimation. Ten different ‘big’ species were selected based on local availability and abundance (Table 1). For convenience, these will be simply referred to as ‘big’ species. Historically, the fields chosen have been used periodically for haying and some cattle grazing but have otherwise been left relatively undisturbed for at least ten years prior to the start of this study. Neighbour competition within this vegetation type is intense; a previous neighbour-removal study at QUBS [38] showed that target individuals (selected randomly) that had near-neighbours left in place were 75–85% smaller in size (dry mass) at the end of the growing season compared with target individuals that had near neighbours removed.

**Table 1:** Size of target species.

Species	Mean dry mass (g)	Mean lateral extent (cm)	Mean plant height (cm)	Mean size index
*Rudbeckia hirta* L.	4.04	17.4	75.2	−2.169
*Solidago juncea* Aiton.	6.35	21.7	78.5	−1.789
*Erigeron philadelphicus* L.	7.74	27.6	71.2	−1.764
*Asclepias syriaca* L.	20.60	18.8	88.8	−1.405
*Aster umbellatus* Miller	6.96	29.5	96.0	−0.778
*Daucus carota* L.	4.94	21.7	109.2	−0.551
*Verbascum thapsus* L.	76.79	29.8	86.1	−0.474
*Cirsium vulgare* (Savi) Tenore.	45.09	25.8	130.3	0.854
*Solidago canadensis* L.	116.88	39.8	121.0	1.804
*Centaurea jacea* L.	400.66	79.8	119.0	6.154

= 3 replicates) dry mass, lateral extent, plant height, and size index for the ten target species, listed in order of increasing size index. Nomenclature follows Gleason and Cronquist (1991). Mean (N

### Defining the Individual

A target individual, and a resident individual counted within a target or random neighbourhood (see below), was defined as a single rooted unit – i.e. the point of transition between an above ground shoot and below ground tissue (or collection of converging shoots that are all attached at or slightly below ground level) [31]. If rhizomatous (or with spreading sprouting roots), the individual was still considered a single rooted unit, despite that it may be connected below-ground to other rooted units nearby.

### Selection of Large ‘Target’ Individuals

**Figure 1:**
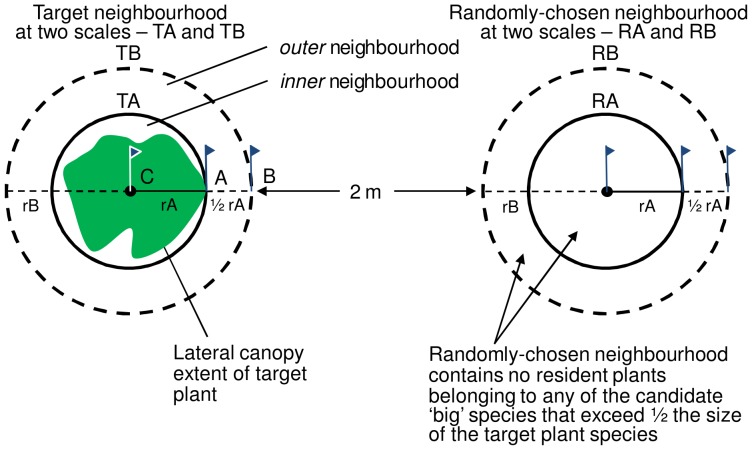
Diagram illustrating the sampling methods used. For the target plot (left), TA represents the inner neighbourhood of radius A (rA), determined as the extreme lateral extent of the target species. TB is extended 0.5 times rA, extending beyond the area in TA. An identically sized random plot, separated by 2 metres from the target neighbourhood was located randomly and had the same dimensions as the associated target neighbourhood.

Sampling was restricted to relatively uniform parts of the site in terms of topography to maximize the chance that the Target neighbourhood and Randomly-chosen neighbourhood (Fig. 1) did not differ noticeably in this respect. Target size was assessed based on visual estimation of above-ground biomass, taking account of both height and lateral extent; i.e. the largest individuals were not necessarily the tallest.

The objective was to assess the effect that a relatively large *individual* (not a clump of individuals) of a particular ‘big’ species has on the composition of its immediate neighbourhood. Accordingly, dense clumps of large plants belonging to the target species were avoided and target neighbourhoods generally contained few or no other members of the target species. The three largest target individuals of each of the chosen ten species within the population were selected, but with the proviso that the target individual could not have any other big individuals of any species as near neighbours. In other words, within both the inner (TA) and outer (TB) target individual neighbourhood (see Fig. 1), there were no other resident plants belonging to any other species that were any larger than half of the size of the target individual–based on visual estimation, and taking both height and lateral extent into account. This was done in order to ensure that potential effects on the composition of the resident species within the target neighbourhoods could not be attributed to any other relatively large plants nearby, i.e. other than the target individual. If this condition was not satisfied, then the potential target individual was rejected for sampling, and the next largest potential target individual available within the local population was chosen. The target eventually chosen, however, was no smaller than ¾ of the size of the largest individual plant in the local population (based on visual estimation).

### Delineation of Target Neighbourhood (Fig. 1)

The target individual’s immediate neighbourhood was defined at two scales, delineating an ‘inner’ versus ‘outer’ target neighbourhood:

TA – the circular area defined by a radius rA centered on the rooted location of the target (C) and extending to the outermost limit of the lateral leaf/branch canopy extent (point A);

TB – the circular area defined by a radius rB = (rA+½ rA), extending to point B, centered on the rooted location of the target (C; Fig. 1).

### Delineation of the Randomly-chosen Neighbourhood (Fig. 1)

Each random neighbourhood was defined at two scales with the same dimensions as its associated target neighbourhood:

RA – the circular area defined by radius rA, centered at a distance of (2 m+rB) away from the perimeter of TB, but otherwise located randomly. The random direction was chosen by using a random number table to generate a compass direction along an arc with the same center as TB (at C).

RB – the circular area defined by radius rB with the same center as RA.

If RA or RB contained any resident plants of any of the candidate ‘big species’ under consideration (including of course the target species in the associated Target neighbourhood) that were greater than ½ the size of the target individual in the Target neighbourhood, then the randomly chosen neighbourhood was rejected. In this case a Random neighbourhood was re-selected at the nearest position that meets the above criterion along the above arc (centered on point C, Fig. 1). This was done in order to ensure that the random neighbourhood did not have a relatively ‘big’ individual of one of the target species, thus creating potentially similar effects to those imposed by the ‘big’ target individual within the target neighbourhood.

### Data Collection

For each of the three target individuals selected for each of the 10 big species, data collection involved 6 stages:

- When the target species was in the flowering stage (with visible open flowers, but before any flowers/dry mass was lost), a suitable target individual (see criteria above and Fig. 1) was located and its height (from ground level to the point of highest reach of plant tissue with the plant standing naturally) and lateral extent (distance from the rooted location to the furthest reaching outer shoots) was recorded. Flags A and B (see Figure 1) were inserted and then the target individual was cut at ground level and placed in a paper bag for later dry-weight measurement in the lab. A flag (C, Fig. 1) was placed where the target individual was rooted; then flag B was inserted and the radius rA and radius rB were recorded (see above and Fig. 1).
- The perimeter for the two scales of the Target neighbourhood was delineated, TA and TB, as described above (Fig. 1): The radius measurements rA and rB were used to calculate the circumferences (2πr) for TA and TB and these perimeters were marked with large adjustable metal hose clamps. Using three more flags, the position for the randomly chosen neighbourhood was marked (see criteria above and Fig. 1).
- A list of all species residing within TA (the ‘inner’ target neighbourhood) was recorded, with notes indicating, for each species, whether or not at least one of the individuals was reproductive (showing flowers or fruits or evidence of their recent attachment to the plant, e.g. a peduncle).
- A list of all species residing within the donut-shaped ‘outer’ target neighbourhood (Fig. 1) was recorded, with notes again indicating, for each species, whether or not at least one of the individuals was reproductive.
- The same adjustable hose clamps from (*ii*) were used to delineate the boundaries of the randomly chosen neighbourhood defined by RA and RB, using the method described above (see Fig. 1).
- Methods (iii) and (iv) were repeated within the randomly chosen neighbourhood–both ‘inner and ‘outer’, defined by RA and RB (See Fig. 1).

### Calculation of the ‘Size Index’

For each target individual, a ‘size index’ was calculated to give equal combined weight to three size-metrics for plant body size: above-ground dry mass, above ground lateral extent (rA in Fig. 1), and height. To do this, we first standardized each of the three measures of size to have a mean of zero and unit variance. The size index was then calculated by adding the three standardized metrics together, creating an index ranging between negative and positive values, with larger positive values indicating a larger plant body size.

### Data for Resident Neighbourhood Species Body Sizes

**Figure 2:**
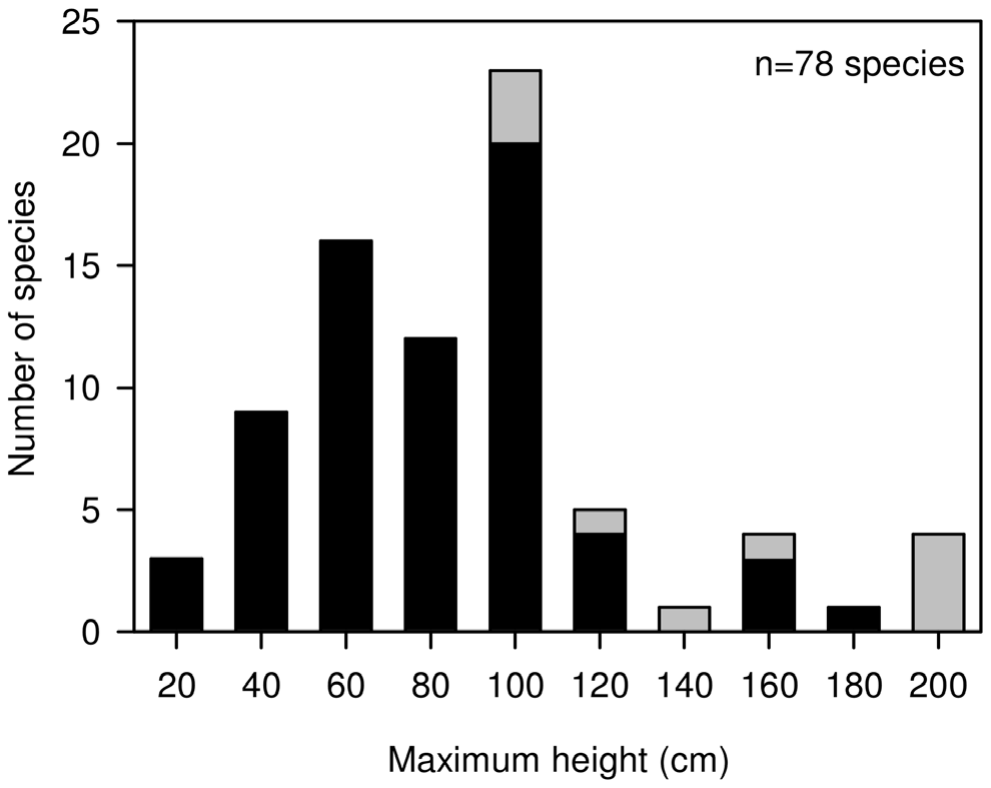
Frequency histogram of the species maximum height. A stacked bar-plot illustrating the distribution of maximum potential heights of community species, taken from Gleason and Cronquist (1991). Black bars represent neighbour species while grey bars represent target species.

The typical maximum plant heights, based on growth in natural conditions, for resident species recorded within target and random neighbourhoods were obtained from published data reported in [39] (Fig. 2). Mean resident species height was calculated for each part of the target and random plots (i.e., inner neighbourhood, outer neighbourhood, total neighbourhood).

### Data Availability

All data used in the analyses presented in this article are available in Table S1.

## Results

### Plant Body Size Index

The three body size metrics (above-ground dry mass, lateral extent, and plant height) were all significantly positively correlated for the three replicates of the ten target species – but still with considerable scatter (height versus lateral extent: r = 0.421, P = 0.02; height versus dry biomass: r = 0.444, P = 0.014; lateral extent versus dry biomass: r = 0.751, P<0.001). Accordingly, since there is no objective basis to favour one metric over the others, calculation of the ‘size index’ allowed each metric to have equal weight for comparing the relative body sizes of the target species.

### Do Large Species Limit Diversity in the Surrounding Neighbourhood?

**Figure 3:**
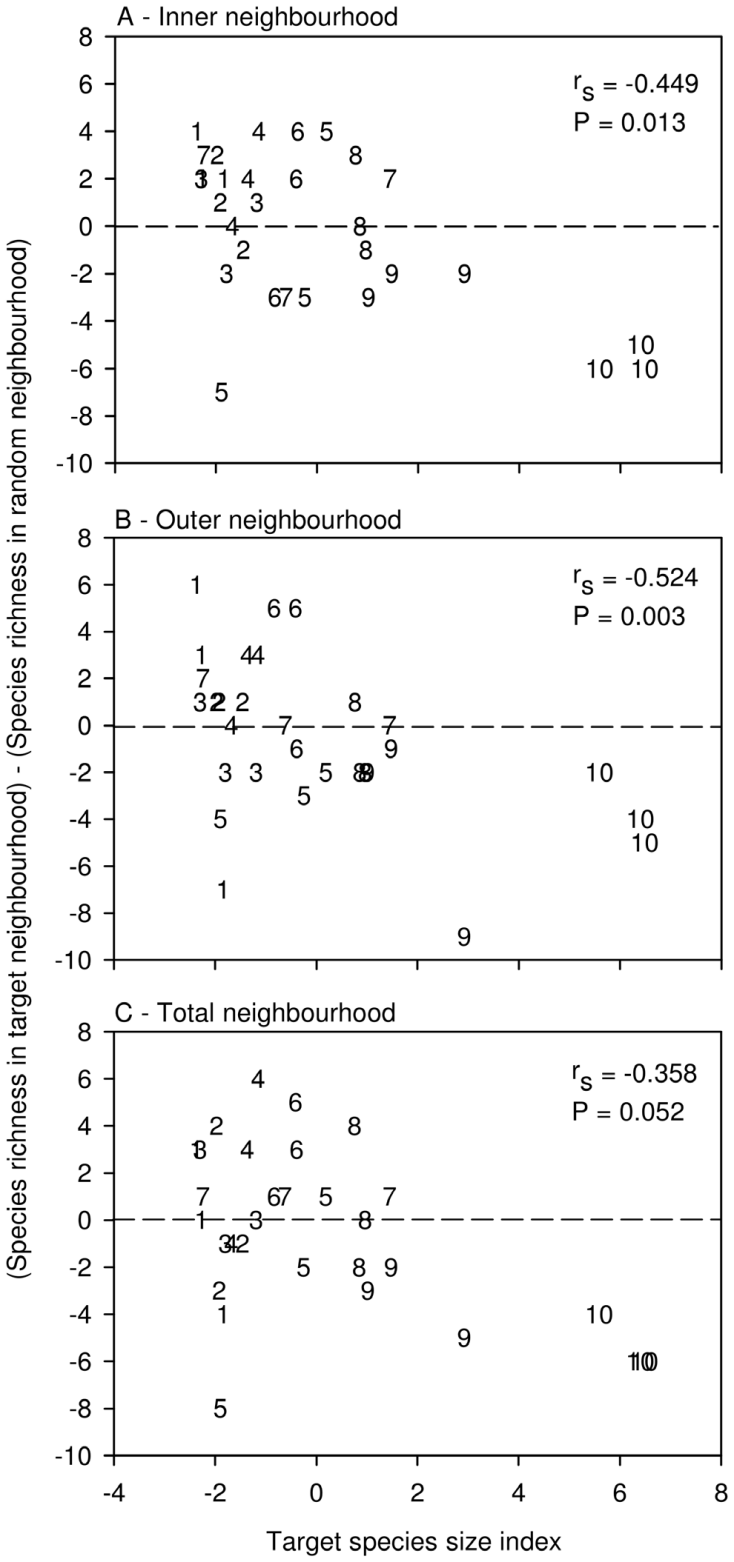
Effects of large target individuals on neighbour plant species diversity. Relationship between target species size index and [(species richness in target neighbourhood) (species richness in random neighbourhood)] for: (a) inner neighbourhoods only; (b) outer neighbourhoods only; and (c) total neighbourhoods. The 3 replicates of each of the 10 target species are coded by numbers 1–10 in order of increasing average species size index: 1- *Rudbeckia hirta*, 2- *Solidago juncea*, 3- *Erigeron philadelphicus*, 4- *Asclepias syriaca*, 5- *Aster umbellatus*, 6- *Daucus carota*, 7- *Verbascum thapsus*, 8- *Cirsium vulgare*, 9- *Solidago canadensis*, 10- *Centaurea jacea*. Dashed line indicates where species richness in target neighbourhood = species richness in random neighbourhood. rs and associated P-values are from Spearman Rank correlation analyses. With *Centaurea jacea* (species 10) omitted,(a) rs =  −0.366, P = 0.062, n = 27; (b) rs = −0.376, P = 0.0533, n = 27; (c) rs = −0.143, P = 0.472, n = 27.

Target species size was negatively related to the difference in species richness between target and random neighbourhoods when analyses focused on the inner neighbourhood (rA; Fig. 3a, P = 0.004) and the outer neighbourhood (rB; Fig. 3b, P = 0.005); however, this correlation was only marginally significant for the total neighbourhood (rA+rB; Fig. 3c, P = 0.053). This means that the degree to which target neighbourhoods contained fewer species than their associated random neighbourhood was influenced by the size of the target individual. However, the influence of target size was very strongly driven by the inclusion of the largest target species, *Centaurea jacea*, in the analysis (without *C. jacea replicates*: inner neighbourhood, P = 0.062; outer neighbourhood, P = 0.053; total neighbourhood, P = 0.472). This negative relationship is further eroded when the fourth largest individual plant, a member of *Solidago canadensis* L. that was also substantially larger than the remaining target individuals, was excluded (inner neighbourhood, P = 0.303, outer neighbourhood, P = 0.378, total neighbourhood, P = 0.991).

### Do Neighbourhoods Centred by a Large Species Contain Fewer Reproductive Species?

**Figure 4:**
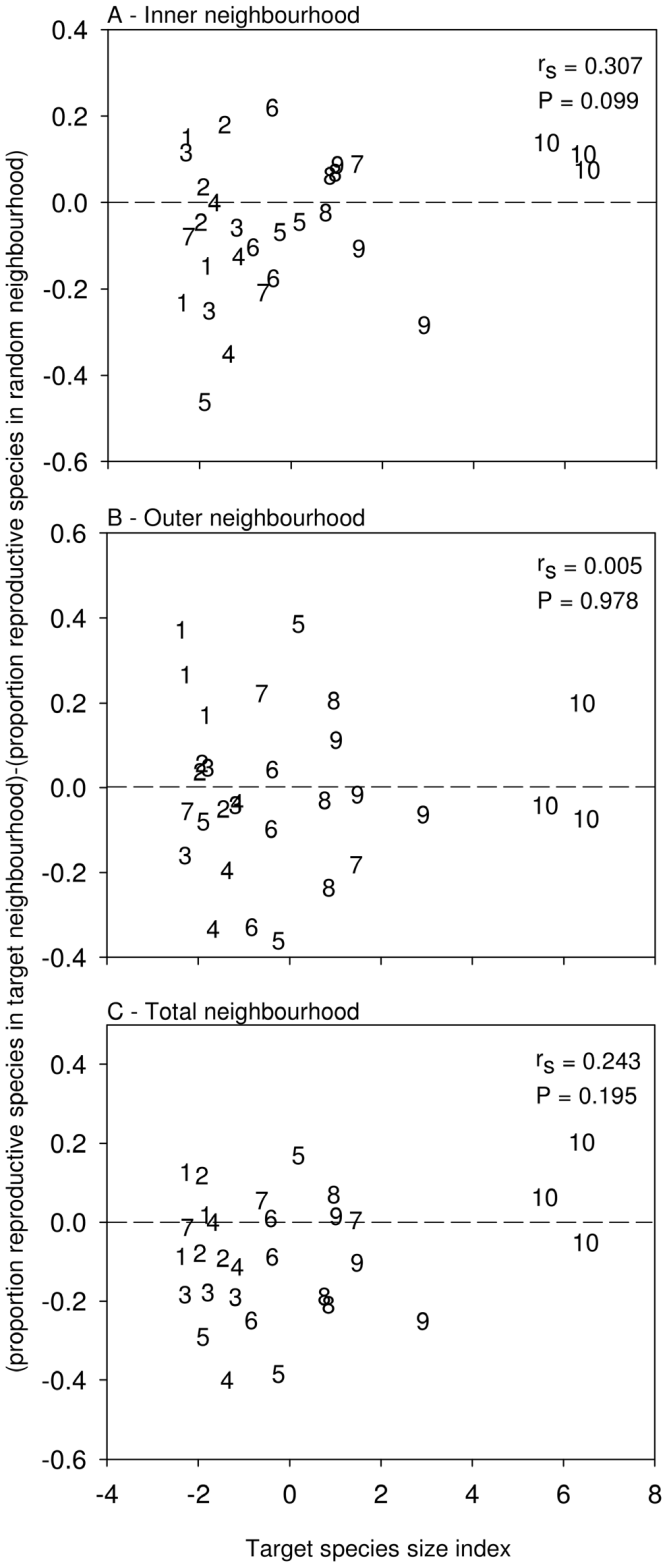
Effects of target individual size on neighbour plant species reproduction. Relationship between target species size index and [(proportion of reproductive species in target neighbourhood) (proportion of reproductive species in random neighbourhood)] for (a) inner neighbourhoods only; (b) outer neighbourhoods only; and (c) total neighbourhoods. The 3 replicates of each of the 10 target species are coded by numbers 1–10 in order of increasing average species size index: 1- *Rudbeckia hirta*, 2*- Solidago juncea*, 3- *Erigeron philadelphicus*, 4- *Asclepias syriaca*, 5- *Aster umbellatus*, 6- *Daucus carota,* 7- *Verbascum thapsus*, 8- *Cirsium vulgare*, 9- *Solidago canadensi*s, 10- *Centaurea jacea*. Dashed line indicates where species richness in target neighbourhood = species richness in random neighbourhood. r_s_ and associated P-values are from Spearman Rank correlation analyses.

There was no decline in the proportion of reproductive species in target neighbourhoods relative to in associated random neighbourhoods (measured as the difference between the two). The proportion of reproductive species in target plots relative to random plots did not change significantly with target species size (Fig. 4a–c, all P>0.05). This lack of relationship was maintained with or without the inclusion of the largest target species, *C. jacea* (all P>0.05; results not presented).

### Do Neighbourhoods Centred by a Large Species Contain Smaller Species?

**Figure 5:**
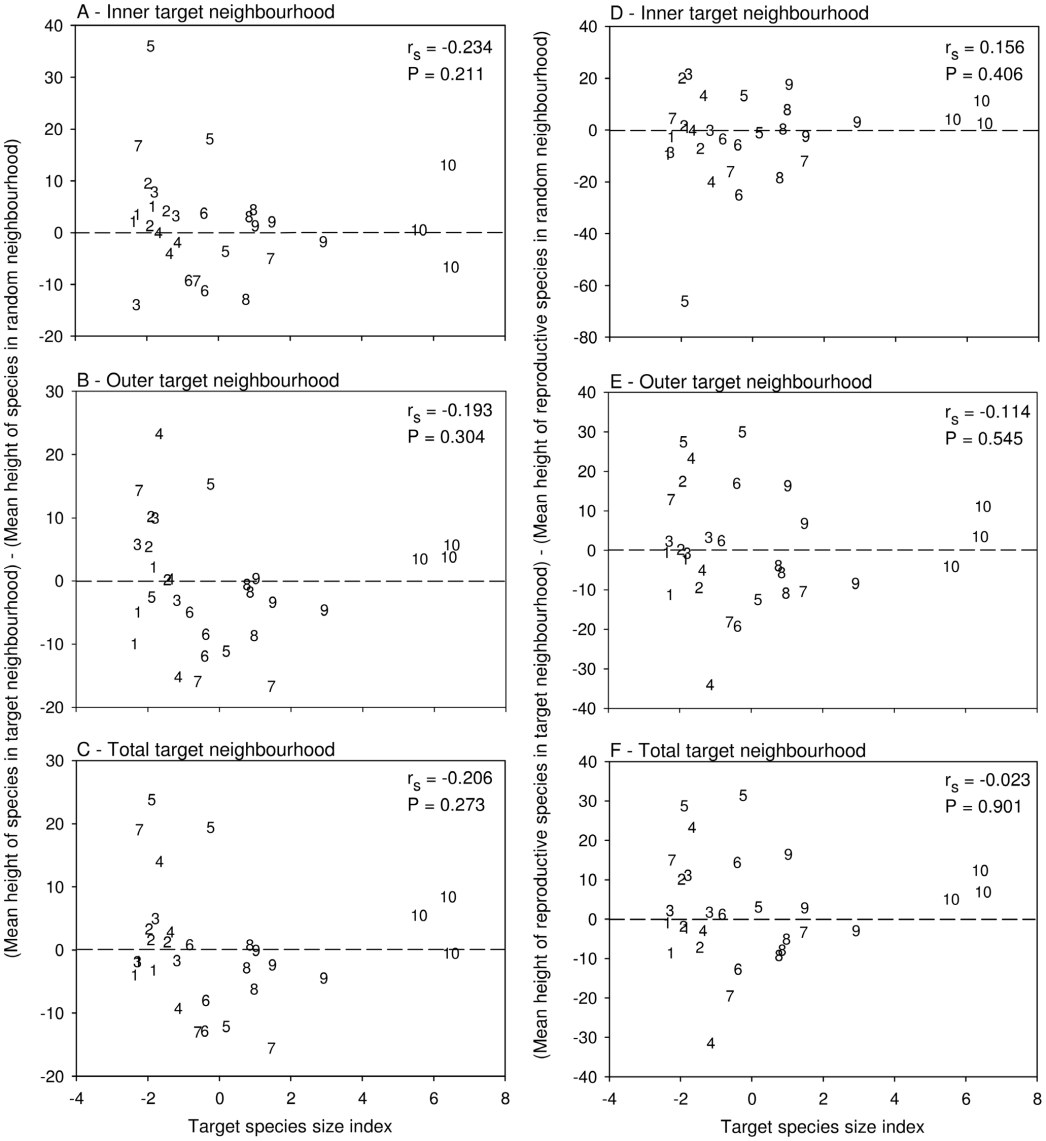
Effects of target individual height on mean neighbour plant species height. Relationship between target species size index and (mean height of species in target neighbourhood-mean height of species in random neighbourhood) (species height is the typical maximum from published data) for: (a) inner target neighbourhoods only; (b) outer target neighbourhoods only; and (c) total target neighbourhoods. The same relationship is also depicted for only reproductive species in target and random neighbourhoods (d-f). The 3 replicates of each of the 10 target species are coded by numbers 1–10 in order of increasing average species size index: 1- *Rudbeckia hirta*, 2*- Solidago juncea*, 3- *Erigeron philadelphicus*, 4- *Asclepias syriaca*, 5- *Aster umbellatus*, 6- *Daucus carota,* 7- *Verbascum thapsus*, 8- *Cirsium vulgare*, 9- *Solidago canadensi*s, 10- *Centaurea jacea.* Dashed line indicates where species richness in target neighbourhood = species richness in random neighbourhood. r_s_ and associated P-values are from Spearman Rank correlation analyses.

The size of target species was not significantly correlated with the difference–between target and associated random neighbourhoods– in mean height of resident plant species (Fig. 5a–c, all P>0.05). We repeated this analysis, focusing on only species within neighbourhoods that were reproducing, and found the same result; target species size did not significantly predict the difference in mean height of reproductive species in target relative to random neighbourhoods (Fig. 5d–f, all P>0.05).

## Discussion

### Do Large Species Limit Diversity in the Surrounding Neighbourhood?

Our primary test of the competitive influence of larger plants on neighbourhood species was to examine the difference in richness between plots with a large focal species (target plots: Fig. 1) and an associated plot of identical size that was not centred by a large focal species (random neighbourhood), in relation to the size of the target individual. Target individual size was significantly negatively correlated with the difference in richness between target neighbourhoods and associated random neighbourhoods; this was true for both the inner neighbourhood and outer neighbourhood, but only marginally so for the total neighbourhood (Fig. 3). However, for all three neighbourhoods, this negative relationship was greatly reduced (P>0.05) after the exclusion of the three replicates of the single largest target species, *C. jacea*, and disappeared completely when the next largest plant, a particularly large *S. canadensis* target, was removed.

The four largest target individuals considered, especially *C. jacea*, are the primary drivers of the observed correlation between target individual size and the difference between species richness in target vs. random neighbourhoods (Fig. 3). *Centaurea jacea* is considerably larger than targets of all other target species (dry biomass: 278% larger than the next largest species; lateral extent: 49.8% larger than the next largest species), although it is only third tallest among the ten target species used (Table 1). The observed patterns are consistent with a size-advantage when the larger competitor is much greater than that of other competitors (i.e., competition is extremely size-asymmetric; [44], [50]. However, this apparent size-advantage is restricted to extreme size differences between the target individual and neighbour species; consistent with other observations that a size-advantage operates on a narrow range of size differences among competitors [36], far narrower than that range of sizes that have been found important in several competition experiments [4], [6], [8], [11], [12]. Importantly, considerable variation in size among plant species does not appear to contribute to the competitive exclusion of smaller neighbour species.

It is possible that the substantial impact of including *C. jacea* results from not only its substantial size, but from other characteristics. For example, it is has been demonstrated that allelochemicals can contribute to the competitive effects of one species on another [40] and it is known that *C. jacea* produces several antriproliferative compounds [41]; although it is not clear whether these chemicals affect the growth of neighbouring plants or function as deterrents to herbivores. Several congeners are known to produce allelochemicals (*C. diffusa* [42], *C. maculosa* [43]). Given that our results remain marginally significant for two scales even when *C. jacea* is removed, and are also very sensitive to the inclusion of the fourth largest target individual, it is more likely that we are observing a size-advantage that is limited to circumstances when the size difference among competitors is extreme.

It is further possible that the asymmetric element of any size-advantage will produce a non-linear relationship [44]. We used a post-hoc partial F statistic to test whether more variation in the difference between target and associated random neighbourhood richness is explained when the quadratic term for size is added to the analysis [45]. According to this analysis, relationships were linear for the inner and outer neighbourhoods, but non-linear for the total neighbourhood (DF = (1,28), Fcrit = 2.89; rA: F = 0.981; rB: F = 0.173; rA+rB: F = 4.54, r^2^ = 0.442, P<0.001). Regardless of the nature of these relationships, it is clear that a size-advantage in this community, insofar as it impacts neighbourhood plant species richness, is limited to the largest of the large plant species in this particular old-field community. Interestingly, this size advantage increases with increased target size regardless of the fact that all targets are above the surrounding canopy. This is not consistent with a previously observed saturation of a size-advantage [44], suggesting instead that the larger a species grows, the greater capacity it has for restricting light to smaller species growing nearby.

Size variation among target species in the study community is, of course, not fully representative of the size variation found in all plant communities. Therefore, it remains possible that in communities with greater species-level size variation, such a size-advantage may play a more important role, particularly given the patterns observed in Fig. 3 when *C. jacea* is included in analysis. Additionally, some noise in these relationships may result from the equal weighting of each of the three measures of plant size when there is a strong possibility that the measures contribute differently to competitive effects. Future work should be directed at understanding what aspect of size is most important in above-ground competition.

It is possible that target neighbourhoods did not contain fewer species than associated random neighbourhoods in general because species in target neighbourhoods benefited from clonal integration [46]. Indeed, increased clonality is generally understood to be associated with smaller species [31]. In particular, our results could be impacted by species that have either stolons or rhizomes such that competitively stressed shoots within the target neighbourhood could be supported by connected shoots outside of that neighbourhood. In light of this, we conducted a post-hoc test to determine whether the number of species in target neighbourhoods with the potential to produce rhizomes or stolons was greater than in associated random neighbourhoods, using paired t-tests. Target neighbourhoods contained no more species capable of producing stolons or rhizomes than did their associated random neighbourhoods (rA: t = −1.16, P = 0.259; rB: t = −1.12, P = 0.273; rA+rB: t = −1.59, P = 0.125). It is worth noting that for each of these analyses, the mean number of species capable of producing stolons or rhizomes was greater in random neighbourhoods than in related target neighbourhoods. Thus, the lack of a general competitive effect of large target individuals on neighbourhood species richness is not clearly driven by clonal integration. Of course, species capable of producing stolons or rhizomes have not all necessarily done so, leaving the possibility that clonal integration may still have played a role in our results. Finally, our measure of size focuses on the ramet, ignoring possible clonal integration for these species. Clonal integration isn’t likely to have impacted our measure of size for target species as only three of ten target species produce stolons or rhizomes, and the choice of targets with few or no conspecific neighbours (see Methods) further reduces the likelihood that targets were clonally integrated. However, the assessment of an above-ground competitive affect should not be impacted regardless, as below-ground competition either is size-symmetric [37] or also advantageous for a larger species [15]. While unlikely, it is possible that resource sharing among ramets for three target species may have added noise to our measure of target individual size.

### Do Neighbourhoods Centred by a Large Species Contain Fewer Reproductive Species?

Target species size was uncorrelated with the proportion of reproductive species in target relative to random neighbourhoods (Fig. 3), a result that was unchanged regardless of the inclusion or exclusion of *C. jacea* data. This result indicates that larger species do not limit the number of reproductive species growing around them in our study community. Importantly, we have not measured the total number of reproductive plants growing in target plots, which leaves open the possibility that large plants do limit reproduction in local neighbourhoods, which could eventually lead to broader changes in the abundance distribution in the community. However, if such changes occur, based on our results, they have not yet occurred in this community. Given that this site has been relatively undisturbed for decades, we believe it is unlikely that a slower loss of small species is taking place; stronger evidence of this would have come from the more coarse consideration of changes in richness and the richness of reproductive species that we have conducted here. Because random neighbourhoods differed in composition from the related target neighbourhood, it was not reasonable to conduct this test using abundance data with this design. It would be difficult to distinguish what proportion of individuals should be reproducing in plots given that nothing is known about the age distribution of plants in target or random plots. Also, given the potential for differences between target species in sizes of their target and random neighbourhoods, any differences in the number of individuals reproducing could be related to differences in total plant density and the size distribution of species within these plots [33], [47].

Regardless of the potential limitation outlined above, our results provide no evidence supporting the suggestion that larger plant size contributes to competitive suppression that reduces reproductive potential in smaller neighbouring plant species, even when we include the extremely large target species *C. jacea.* One explanation for this result is that small species have evolved greater reproductive economy, promoted by a relatively small minimum reproductive threshold size and/or the capacity to reproduce at a small fraction of their mature size [31], [48]. Recent evidence indicates that the efficiency with which plants convert biomass production to reproduction decreases with increasing plant size [49], [50]. Thus, even if large plants have the capacity to restrict the growth of neighbouring smaller plants, the latter may still reproduce. A related possibility is that large species, through their relatively inefficient use of local resources, leave both space and resources available for the successful growth of small species with an associated small physical space niche [16], [32].

### Do Neighbourhoods Centred by a Large Species Contain Smaller Species?

To further explore the impact of target plant size on neighbourhoods, we tested whether target size predicted differences in the mean species sizes for those species residing within target neighbourhoods relative to random neighbourhoods. Target plant size had no apparent impact on the difference in mean resident species size in target versus random neighbourhoods (Fig. 4). This was true whether the analysis focused on all neighbour species (Fig. 4a–c), or only on reproductive neighbouring species (Fig. 4d–f). This indicates, therefore, that larger target plants are not more likely to exclude smaller species from their local neighbourhoods, contrary to the ‘size-advantage’ hypothesis [34]. This result also contrasted with our prediction that small species, potentially benefitting from small and diverse physical space niches generated by large target individuals, would be over-represented in target neighbourhoods.

In general, little evidence has accrued from field studies in support of the prediction that large plant species have a consistent competitive advantage over smaller species. For example, several studies have produced no evidence that coexisting species are convergent with respect to plant species size [33], [34], [51]. However, one study has found evidence that similar sized species are more likely to be found coexisting [52] and another found evidence that species differing in maximum height coexisted more often than expected by chance [53]. This collection of studies does not support the notion of a clear size-based competitive effect in natural systems. Our current findings are also consistent with work demonstrating that as mean plant species size increases along a dune succession, variation in the size of neighbours was not reduced as succession proceeded, as would be predicted under a traditional “size-advantage” hypothesis [35].

As with the analysis of the number of species reproducing in target versus random neighbourhoods, our analysis of the species size distribution for neighbourhoods does not incorporate abundance data. Thus it remains a possibility that small species are under-represented within plots centred by a large target plant, although it also remains a possibility that smaller species, represented by smaller reproductive plants may be over-represented [48]. Regardless, this analysis clearly indicates that at the species level, there is no evidence that larger target plants significantly impact the size distribution in the local neighbourhood, a logical prediction from traditional theory predicting a size-advantage in competition.

### Conclusions

Neighbourhoods centred by a large target plant contained fewer neighbour species than associated random neighbourhoods of the same size (Fig. 2); however, this pattern was driven by the four largest individuals in the analysis, including all three samples of a single large species, *C. jacea*. The size of a target individual did not have a significant effect on the proportion of species reproducing (Fig. 3), or the species-size distribution (Fig. 4), in the local neighbourhood compared with random neighbourhoods. While extremely large target individuals were found to reduce neighbourhood plant richness, overall, we found limited support for a general size-advantage, as has been suggested in several simple, short-duration competition experiments [4], [11], [12]. The growing evidence that such a size-advantage either does not act, or is relatively weak, in natural vegetation signals the limited ability to extrapolate from the results of such competition experiments. The fact that increased body size does not confer a clear advantage emphasizes the need going forward to establish just what conditions favour large plant size, and therefore larger plant species. It remains interesting that many competition experiments have generally supported such an advantage while field studies have generally not. Possible explanations for this disconnect include the general use of biomass based measures of performance [19] which is likely an inadequate measure of fitness [20], the likelihood that smaller species benefit from greater reproductive economy [21], [31], [50] or are better at occupying resources not captured by large species [32], or that competition in natural communities is predominantly below-ground and size-symmetrical ( [37], but see [15]). Clonal integration does not appear to explain a lack of size-advantage, but warrants further research.

## Supporting Information

Table S1 Data used to analyze the impact of large target plants on local neighbourhoods.
(DOCX)
