# Supplementary material for: Effects of ‘Target’ Plant Species Body Size on Neighbourhood Species Richness and Composition in Old-Field Vegetation
Source: PLoS One. 2013 Dec 13;8(12):e82036. doi: 10.1371/journal.pone.0082036 (PMC3862571; doi:10.1371/journal.pone.0082036)
Supplement: Table S1 — Data used to analyze the impact of large target plants on local neighbourhoods. (DOCX) [file pone.0082036.s001.docx]

| Table S1: All data used in analyses for this paper are presented in the table below, with column explanations presented on the following page | | | | | | | | | | | | | | |
| --- | --- | --- | --- | --- | --- | --- | --- | --- | --- | --- | --- | --- | --- | --- |
|  |  |  |  |  |  |  |  |  |  |  |  |  |  |  |
|  |  |  | Species Richness | | | Proportion Reproductive | | | Mean Species Potential Height (All) | | | Mean Species Potential Height (Reproductive) | | |
| Species | Replicate | Size | Ta-Ra | Tb-Rb | Ttot-Rtot | Ta-Ra | Tb-Rb | Ttot-Rtot | Ta-Ra | Tb-Rb | Ttot-Rtot | Ta-Ra | Tb-Rb | Ttot-Rtot |
| Rudbeckia hirta | 1 | -2.2792 | 2 | 0 | -1 | 0.1518 | 0.2667 | 0.127 | 4.1518 | -3.75 | -1.7647 | 5 | -7.5974 | 7.0455 |
|  | 2 | -1.8477 | 2 | -7 | -4 | -0.1469 | 0.1712 | 0.0178 | -0.75 | -2.4848 | -7.096 | -16.5 | -10 | -11.4286 |
|  | 3 | -2.3805 | 4 | 6 | 4 | -0.2316 | 0.3725 | -0.0863 | -1.8333 | -13.9786 | -7.4135 | -19.2857 | -7.5 | -12.5325 |
| Cirsium vulgare | 1 | 0.9622 | -1 | -2 | -1 | 0.0694 | 0.2054 | 0.0706 | -4.3056 | -4.2857 | -6.1765 | -6 | -0.7937 | 5.7143 |
|  | 2 | 0.7545 | 3 | 1 | 4 | -0.0222 | -0.0294 | -0.1871 | -8.1667 | 3.3333 | 0.7083 | -8.3036 | 3.8889 | -7.5 |
|  | 3 | 0.8459 | 0 | 0 | 0 | 0.0625 | -0.2353 | -0.2087 | 0.625 | -1.451 | 1.0278 | -5.2679 | -9.6667 | -9.2614 |
| Solidago canadensis | 1 | 1.0154 | -3 | -2 | -3 | 0.0873 | 0.1128 | 0.015 | -0.5714 | 0.5664 | 0.1667 | 17.7273 | 16.3462 | 19.1026 |
|  | 2 | 2.9181 | -2 | -9 | -5 | -0.2832 | -0.0633 | -0.2507 | 0.3784 | -4.6222 | -2.7174 | 3.2059 | -8.4167 | -9.5635 |
|  | 3 | 1.4802 | -2 | -1 | -2 | -0.1059 | -0.0158 | -0.1023 | -0.2118 | -3.2684 | -2.1682 | -5.6944 | 3.9835 | -1.8681 |
| Doellingeria umbellata | 1 | -0.2518 | -1 | -3 | 1 | -0.0682 | -0.3611 | -0.3839 | 23.6883 | 19.8571 | 23.5769 | 13.3333 | 30 | 30 |
|  | 2 | -1.902 | -8 | -3 | -7 | -0.4615 | -0.0795 | -0.2898 | 40.5833 | -6.3 | 16.0381 | -66 | 27.5 | 18 |
|  | 3 | 0.1812 | 6 | -1 | 3 | -0.0444 | 0.3864 | 0.1692 | -0.9583 | -11.3535 | -10.8681 | -1 | -12.5 | -2.5 |
| Erigeron philadelphicus | 1 | -1.7992 | -2 | -2 | -1 | -0.25 | 0.0476 | -0.1778 | 7.9167 | 10 | 5 | 21.6667 | -0.6667 | 12.1429 |
|  | 2 | -1.1945 | 1 | -2 | 0 | -0.0577 | -0.041 | -0.1889 | 3.4679 | -2.9436 | -1.5333 | 0 | 3.4646 | 2.0227 |
|  | 3 | -2.2993 | 2 | 1 | 3 | 0.1167 | -0.1591 | -0.1833 | -14 | 5.7955 | -1.9667 | -8.5227 | 2.3333 | -6.1111 |
| Solidago juncea | 1 | -1.4651 | -2 | 0 | -2 | 0.1804 | -0.05 | -0.0909 | 7.9451 | 3.25 | 4.0364 | -7.0833 | -9.4444 | -5.6154 |
|  | 2 | -1.9782 | 3 | 1 | 4 | -0.0455 | 0.0375 | -0.0788 | 9.7597 | 5.5708 | 3.4491 | 20.4524 | 0.2381 | 14.625 |
|  | 3 | -1.926 | 1 | 1 | -3 | 0.0364 | 0.0577 | 0.1176 | 1.3818 | 10.2051 | 1.5714 | 1.7857 | 17.5 | -0.9524 |
| Centaurea jacea | 1 | 5.5857 | -7 | -2 | -5 | 0.1389 | -0.042 | 0.0633 | 0.5556 | 3.4965 | 5.5208 | 4.1667 | -4.125 | 5.625 |
|  | 2 | 6.4797 | -7 | -5 | -6 | 0.0758 | -0.0725 | -0.0515 | -6.5714 | 5.7353 | -0.5192 | 2.6538 | 11.152 | 5.609 |
|  | 3 | 6.3975 | -6 | -10 | -12 | 0.1125 | 0.2 | 0.2028 | 13.0833 | 3.8246 | 8.4399 | 11.4359 | 3.5119 | 8.8485 |
| Asclepias syriaca | 1 | -1.6845 | 0 | 0 | -1 | 0 | -0.3333 | 0 | 0 | 23.3333 | 14 | 0 | 23.3333 | 0 |
|  | 2 | -1.3755 | 2 | 3 | 3 | -0.35 | -0.1944 | -0.4 | -4.2 | 0.1111 | 2.6923 | 13.3333 | -5 | 2.1429 |
|  | 3 | -1.1574 | 4 | 3 | 6 | -0.125 | -0.0357 | -0.1136 | -1.625 | -14.9643 | -8.95 | -20 | -34 | -12.5 |
| Verbascum thapsus | 1 | -0.6201 | 2 | 0 | 1 | -0.2083 | 0.2222 | 0.0556 | -9.2857 | -15.9091 | -13.0128 | -16 | -18.1944 | -22.8333 |
|  | 2 | -2.2497 | 4 | 3 | 2 | -0.0779 | -0.0556 | -0.013 | 9.7857 | 7.9798 | 13.2197 | -7.1667 | 5.0694 | 7.2619 |
|  | 3 | 1.4457 | -3 | 0 | 1 | 0.0889 | -0.1818 | 4.81E-03 | -5 | -16.6667 | -15.5556 | -12 | -10.4762 | -5 |
| Daucus carota | 1 | -0.4185 | 1 | 5 | 4 | 0.2206 | -0.0966 | 0.0103 | 6.9375 | -9.35 | -15.3146 | 7.875 | 18.5 | 21.8182 |
|  | 2 | -0.8445 | -5 | 6 | 2 | -0.1029 | -0.3279 | -0.2489 | -10.5909 | 0.4385 | 1.808 | -12.6667 | 5 | 1.6667 |
|  | 3 | -0.3924 | 4 | -3 | 0 | -0.1746 | 0.0431 | -0.0874 | -4.1 | -4.3272 | -3.8647 | -25 | -1.6667 | -7 |

Data Explanation by column:

A. Target species for which size was determined (3 replicates per target species)

B. Replicate - Indicates the replicate number for each target

C. Size index for target species (calculated as the sum of three measures of size, standardized to the mean of zero and unit variance - height, dry above-ground biomass, lateral extent)

D. Species richness in the inner Target neighbourhood (Ta) minus than in the inner neighbourhood of an associated random neighbourhood (Ra), which is the same size but lacks the central target plant

E. Species richness in the outer Target neighbourhood (Tb) minus than in the outer neighbourhood of an associated random neighbourhood (Rb), which is the same size but lacks the central target plant

F. Species richness in the total Target neighbourhood, which combines neighbourhoods a and b (Ttot) minus than in the total neighbourhood of an associated random neighbourhood (Rtot), which is the same size but lacks the central target plant

G. Proportion of neighbour species that are reproductive in the inner Target neighbourhood (Ta) minus than in the inner neighbourhood of an associated random neighbourhood (Ra), which is the same size but lacks the central target plant

H. Proportion of neighbour species that are reproductive in the outer Target neighbourhood (Tb) minus than in the outer neighbourhood of an associated random neighbourhood (Rb), which is the same size but lacks the central target plant

I. Proportion of neighbour species that are reproductive in the total Target neighbourhood, which combines neighbourhoods a and b (Ttot) minus than in the total neighbourhood of an associated random neighbourhood (Rtot), which is the same size but lacks the central target plant

J. Mean species potential height of neighbour species in the inner Target neighbourhood (Ta) minus than in the inner neighbourhood of an associated random neighbourhood (Ra), which is the same size but lacks the central target plant

K. Mean species potential height of neighbour species in the outer Target neighbourhood (Tb) minus than in the outer neighbourhood of an associated random neighbourhood (Rb), which is the same size but lacks the central target plant

L. Mean species potential height of neighbour species in the total Target neighbourhood, which combines neighbourhoods a and b (Ttot) minus than in the total neighbourhood of an associated random neighbourhood (Rtot), which is the same size but lacks the central target plant

M. Mean species potential height of reproductive neighbour species in the inner Target neighbourhood (Ta) minus than in the inner neighbourhood of an associated random neighbourhood (Ra), which is the same size but lacks the central target plant

N. Mean species potential height of reproductive neighbour species in the outer Target neighbourhood (Tb) minus than in the outer neighbourhood of an associated random neighbourhood (Rb), which is the same size but lacks the central target plant

O. Mean species potential height of reproductive neighbour species in the total Target neighbourhood, which combines neighbourhoods a and b (Ttot) minus than in the total neighbourhood of an associated random neighbourhood (Rtot), which is the same size but lacks the central target plant
